# Overview of Hepatitis B Vaccine Non-Response and Associated B Cell Amnesia: A Scoping Review

**DOI:** 10.3390/pathogens13070554

**Published:** 2024-07-02

**Authors:** Nura Bello, Shuaibu A. Hudu, Ahmed S. Alshrari, Mustapha U. Imam, Abdulgafar O. Jimoh

**Affiliations:** 1 Department of Pharmacology and Therapeutics, Faculty of Basic Clinical Sciences, College of Health Sciences, Usmanu Danfodiyo University, Sokoto 840232, Nigeria; bnura@abu.edu.ng; 2 Department of Pharmacology and Therapeutics, Faculty of Pharmaceutical Sciences, Ahmadu Bello University, Zaria 810107, Nigeria; 3 Department of Basic Medical and Dental Sciences, Faculty of Dentistry, Zarqa University, Zarqa 13110, Jordan; 4 Department of Medical Microbiology and Parasitology, Faculty of Basic Clinical Sciences, College of Health Sciences, Usmanu Danfodiyo University, Sokoto 840232, Nigeria; 5 Medical Laboratory Technology Department, Faculty of Applied Medical Science, Northern Border University, Arar 91431, Saudi Arabia; ahmed.alsharari@nbu.edu.sa; 6 Centre for Advanced Medical Research and Training, Usmanu Danfodiyo University, Sokoto 840232, Nigeria; mustapha.imam@udusok.edu.ng

**Keywords:** hepatitis B vaccine, immunogenicity, non-response, lymphocytes, cytokines, amnesia

## Abstract

Background: The advent of the hepatitis B vaccine has achieved tremendous success in eradicating and reducing the burden of hepatitis B infection, which is the main culprit for hepatocellular carcinoma—one of the most fatal malignancies globally. Response to the vaccine is achieved in about 90–95% of healthy individuals and up to only 50% in immunocompromised patients. This review aimed to provide an overview of hepatitis B vaccine non-response, the mechanisms involved, B cell amnesia, and strategies to overcome it. Methods: Databases, including Google Scholar, PubMed, Scopus, Cochrane, and ClinicalTrials.org, were used to search and retrieve articles using keywords on hepatitis B vaccine non-response and B cell amnesia. The PRISMA guideline was followed in identifying studies, screening, selection, and reporting of findings. Results: A total of 133 studies on hepatitis B vaccine non-response, mechanisms, and prevention/management strategies were included in the review after screening and final selection. Factors responsible for hepatitis B vaccine non-response were found to include genetic, immunological factors, and B cell amnesia in healthy individuals. The genetic factors were sex, HLA haplotypes, and genetic polymorphisms in immune response markers (cytokines). Non-response was common in conditions of immunodeficiency, such as renal failure, haemodialysis, celiac disease, inflammatory bowel disease, hepatitis C co-infection, and latent hepatitis B infection. Others included diabetes mellitus and HIV infection. The mechanisms involved were impaired immune response by suppression of response (T helper cells) or induced suppression of response (through regulatory B and T cells). Discussion: A comprehensive and careful understanding of the patient factors and the nature of the vaccine contributes to developing effective preventive measures. These include revaccination or booster dose, vaccine administration through the intradermal route, and the use of adjuvants in the vaccine.

## 1. Introduction

Hepatitis B is one of the major infections contributing to the global burden of disease, with an estimated prevalence of over 350 million people with the chronic form of the disease, characterised by the presence of the surface antigen of hepatitis B (HBsAg) in the blood beyond six months, and resulting in approximately one million deaths annually [1]. It represents the main culprit in the development of liver cancer, also referred to as hepatocellular carcinoma and liver cirrhosis, accounting for about 50% and 33% of mortality, respectively, which is the third leading cause of mortality in cancer after lung and colorectal cancers [2]. In addition, it also leads to fulminant hepatitis on some rare occasions (approximately 1%) [3]. The development of a recombinant vaccine for hepatitis B has yielded significant success in reducing the burden of chronic infection with hepatitis B, especially in countries with wider coverage from childhood to adulthood [4]. The immune response to the hepatitis B vaccine is determined by assessing the concentration of neutralising antibody (anti-HBsAg), and the optimum level required is ≥10 IU/L, though only individuals that produce ≥100 IU/L are considered high responders in the UK, while individuals that produce the antibody within the range of ≥10 IU/L and ≥100 IU/L are regarded as low responders, meaning that their immunity period after vaccination will be less than that of high responders [5,6,7]. Despite the success achieved in the use of the vaccine, an adequate immune response is not achieved in about 5–10% of the population, especially healthcare personnel, and is higher in some vulnerable groups, such as immunocompromised individuals and chronic kidney disease patients on haemodialysis, where up to 50% non-response may occur. Non-response is also common in other conditions, such as celiac disease, diabetes mellitus, hepatitis C infection, use of immunomodulators, and in individuals with some genetic dispositions, etc. [8]. Again, evidence confirms that non-response to the hepatitis B vaccine is not only related to vaccinee-associated factors such as age, sex, obesity, and smoking, but may also depend on the nature of the vaccine, dose, and type of adjuvants used [9,10]. The non-response, if not curtailed, can lead to an enhanced occurrence of chronic hepatitis B infection, resulting in a higher risk of complications as well as transmission. Multiple research studies have been carried out to study the factors contributing to the non-response, ranging from epidemiological factors to defective immunological mechanisms and genetic polymorphisms involving antigen-presenting cells, cytokines, and cytokine receptors. This review is aimed at providing an overview of hepatitis B vaccine non-response: prevalence and occurrence in various disease conditions, mechanisms involved, relationship with B cell amnesia, and approaches to management in healthy and/or diseased population groups.

## 2. Methods

### 2.1. Search

**Figure 1:**
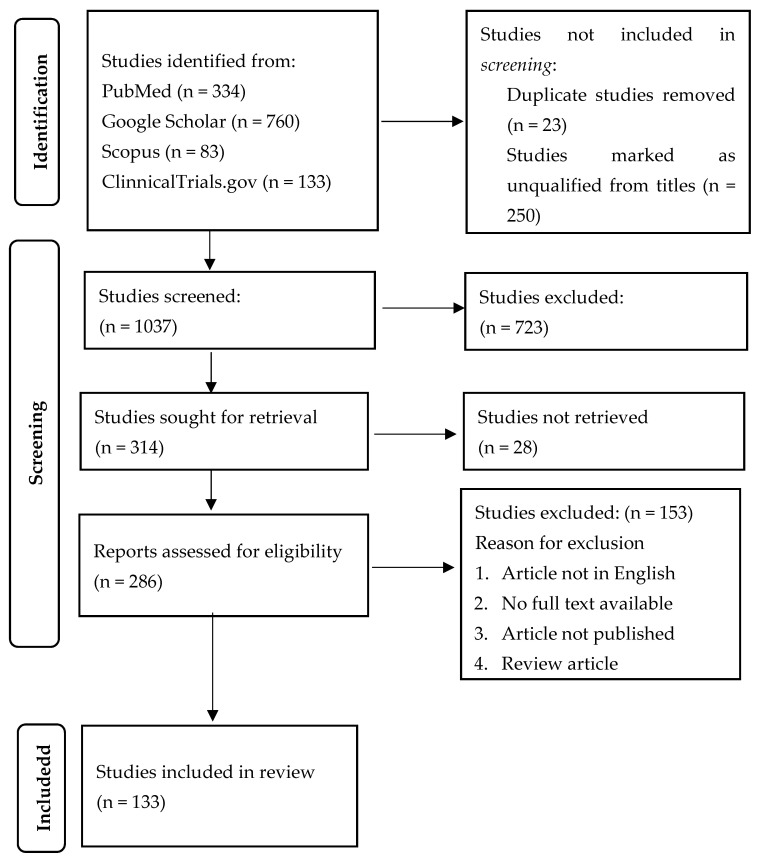
Summary flowchart of identification, screening, and selection of studies for the review.

The search for articles was carried out in the following databases: Google Scholar, PubMed, Web of Science, Cochrane, and clinicaltrials.org, with the keywords: “hepatitis B vaccine”, “non-response/hypo-response/non-responders”, “immunogenicity”, and “B cell amnesia”, using Boolean operators. The studies retrieved were published between 1989 and 2023. In this study, 133 were selected and included after screening and comparing against eligibility criteria. The lists of references of the articles selected were also explored for additional articles (Figure 1).

### 2.2. Eligibility Criteria

Criteria for inclusion included studies carried out in persons who have been vaccinated against the hepatitis B virus; studies that explore the possible causes of immune non-response to the vaccine against hepatitis B and the relationship between the immune non-response and B cell amnesia; studies that investigate potential mechanisms behind non-responsiveness, such as lymphocytes and cytokine expression levels; studies that explore the potential strategies for improving immune response to the vaccine, such as increased vaccination dose, intradermal administration, and the use of adjuvanted vaccines; studies that consider the possible pros and cons of these strategies, and any other factors that may influence the vaccine response.

The exclusion criteria include studies not published in English, studies that do not investigate the causes of immune non-response to the hepatitis B vaccine, studies that do not explore potential mechanisms or approaches to improve the response to the vaccine, or studies that do not provide complete data. Information was extracted from the studies that fulfilled the inclusion criteria using a data extraction form, and the data were synthesised using a narrative synthesis approach.

The protocol for the review was drafted and published in PROSPERO, an international database for prospective registration of systematic review protocols, with registration number: PROSPERO 2023 CRD42023457503, which is now available from: https://www.crd.york.ac.uk/PROSPERO/display_record.php?RecordID=457503 (accessed on 23 September 2023).

## 3. Results

### 3.1. Hepatitis B Vaccine Non-Response Prevalence and Risk Factors

Despite global efforts to reduce the impact of chronic hepatitis B infection through vaccination, which has been made part of the routine childhood immunisation in many countries, failure has been encountered in achieving absolute protection. This failure has been due to increasing reports of immune non-response to the vaccine. Non-response to the hepatitis B vaccine with the normal three-dose series has been reported in many studies within the range of 5–10% or 10–15% within healthy individuals [11,12]. Non-response is caused by a variety of factors, including advanced age, obesity, smoking, chronic illness, and hereditary factors [13]. The risk increases in individuals with other conditions, as explained below.

#### 3.1.1. Chronic Hepatitis C

The presence of chronic hepatitis C infection significantly increases the severity of chronic hepatitis B, leading to a higher risk or actual occurrence of complications (cirrhosis of the liver and hepatocellular carcinoma) and a reduction in the immune response to the hepatitis B vaccine [14]. There was conflicting evidence on the immunogenicity of the hepatitis B vaccine in individuals infected with chronic hepatitis C [15]. Non-response in hepatitis C co-infection is generally higher (10–15%) than in the general population (5–10%) [16]. A recent finding observed that co-infection of hepatitis B with chronic hepatitis C in adults after treatment with direct-acting antiretroviral drugs had a significant non-response rate (43%), which was also associated with advanced age and the occurrence of isolated HbcAb or latent hepatitis B infection [17]. The mechanisms contributing to the immune non-response to the hepatitis B vaccine in hepatitis C include the involvement of Tim-3 (T cell immunoglobulin mucin domain-3) produced by monocytes, which is upregulated, resulting in an imbalance of IL-12/IL-23 (IL-12 inhibition and IL-23 enhancement) in the innate immune system and, consequently, accumulating Th17 in the adaptive immune system [18]. Another finding also confirmed the involvement of the programmed death receptor-1 (PD-1), usually expressed on the T helper cells, which is usually upregulated in chronic hepatitis C infection, thereby interfering with the function of lymphocytes in responding to the HBV vaccine due to dysregulation [19]. 

#### 3.1.2. Chronic Kidney Disease (CKD) and Haemodialysis

CKD and haemodialysis patients are well known to be more vulnerable to complications of hepatitis B infection, such as cirrhosis and hepatocellular carcinoma, as they are highly susceptible to hepatitis B infection, likely due to increased administration of blood products in haemodialysis, contamination of equipment used for dialysis, and other physiological and environmental factors [20]. The risk is usually reduced with routine vaccination and effective haemodialysis [21,22]. Factors contributing to non-response, especially in haemodialysis, are multiple in number [23]. Some studies have observed a link between non-response in haemodialysis patients with conditions including hepatitis C, obesity, diabetes mellitus, nutrition, advanced age, and duration of haemodialysis [24]. IL-12, a potent regulatory cytokine that plays a significant role in hepatitis B virus clearance, was observed to be relatively lower in non-responders with chronic kidney disease undergoing dialysis [25]. Also, a significant interaction was observed using “chi-square automatic interaction detection” between gender, dose of vaccine, and dialysis frequency, with gender (male) being most important, especially when combined with a higher dialysis frequency [26]. Studies performed in patients with chronic kidney disease also identified a link between non-response to the hepatitis B vaccine and deficiency in vitamin D (both 25-hydroxycholciferol and 1,25-dihydroxycholecalciferol), as it plays a role in regulating the production of cytokines and enhancing the antimicrobial capacity of monocytes, neutrophils, and natural killer cells [27]. Also, a study on single-nucleotide polymorphisms of the vitamin D system, which include vitamin D receptor, vitamin D binding protein, and retinoic X receptor alpha, in chronic kidney disease patients associated only the rs1544410 polymorphism in the vitamin D receptor with non-response to the hepatitis B vaccine, which is more common with homozygotes “AA” and less common with homozygotes “GG” [28].

#### 3.1.3. Celiac Disease

Hepatitis B vaccine non-response is significantly higher (50–70%) in celiac disease patients compared to normal individuals (5–10%) [29,30,31]. This makes them more susceptible to chronic hepatitis B infection depending on the patients’ age category, and level of diagnosis, and treatment [32]. The non-response has been associated with competition between HbsAg and glacial peptides in binding to HLA-DQ2, HLA-DQ8, HLA-DR3, and HLA-DR7 haplotypes, which leads to impairment of the response through antigen presentation, proliferation of T lymphocytes, and production of HbsAg-specific antibodies [33]. In these patients, the response may be improved by enhanced compliance with gluten-free diets and the administration of booster vaccinations [30,34,35].

#### 3.1.4. Latent HBV Infection

Luo et al. [36] first performed a study in which non-responders to the hepatitis B vaccine were assessed and HBV DNA was detected in about 60–70% of them, indicating the presence of a latent HBV infection that may induce tolerance to the vaccine through immunosuppression, enhancement of the regulatory T cell activity, or inhibition of the response of B and T lymphocytes, as reported in other studies [37,38]. This is also referred to as “occult HBV infection”, which occurs after serorecovery from the infection with the maintenance of specific T cell activity that prevents reactivation [39]. Meanwhile, another finding indicated the reactivation of fulminant hepatitis B infection due to inflammatory bowel disease and treatment with infliximab [40,41].

#### 3.1.5. Inflammatory Bowel Disease

Inflammatory bowel disease is a condition generally characterised by a state of immunosuppression as a result of using immunomodulators (corticosteroids, thiopurines, monoclonal antibodies, etc.) for treatment and the negative effect of the disease on the activity of the immune system. In addition, it involves many risk factors for transmission of hepatitis B virus, such as continuous blood transfusion, colonoscopy, surgery, and other parenteral medications [42]. As such, hepatitis B vaccination is included in the routine management of inflammatory bowel disease patients based on different standard guidelines [43]. Despite that, hepatitis B vaccine non-response has been observed to occur in about half of the vaccinated patients with IBD, likely due to the reasons listed above, which are more compounded in older patients [44,45,46]. Consequently, having considered non-response categories, new formulation types with special adjuvants, booster vaccination, and dose adjustment can be relatively effective in addressing the issue in this patient group [8,47]. Other conditions in which immunosuppressants are used, such as psoriasis (Th1 associated), atopic dermatitis (Th2 associated), and morphea, are highly susceptible to chronic infection, likely due to the nature of the disease condition and the drugs used [48]. Therefore, testing before the commencement of therapy is essential, as is the need for booster vaccination if needed. 

#### 3.1.6. Other Conditions

The immune response to the hepatitis B vaccine in diabetes, especially insulin-dependent diabetes mellitus (IDDM) in children, has been investigated and reported to be lower (average 58%) compared to their healthy counterparts (average 90%) [49,50]. This was in agreement with the report in a systematic review and meta-analysis confirming a generally weaker immune response to the hepatitis B vaccine in diabetics [51,52]. The non-response occurs as a result of the effect of diabetes on various components of the immune system, ranging from antigen processing and presentation to suppression of B cell responses. The presence of the DR3/DQ2 and DR4/DQ8 HLA haplotypes in about 90% of diabetics is also a strong marker of hepatitis B vaccine non-responsiveness [53]. HIV infection is also among the conditions in which HBV vaccine non-response is common. The prevalence among HIV patients has reached up to 44–76%, contrary to 5–10% in healthy people [54]. The major culprit for the non-response was a decreased level of CD4 T cells. Other cellular and molecular mechanisms include the upregulation of regulatory T cells (Tregs), leading to the release of IL-10, and subsequently deregulating the function of T and B cells through a complex mechanism [55,56].

### 3.2. Mechanisms Involved in Hepatitis B Vaccine Non-Response

#### 3.2.1. Pattern Recognition Receptors

Pattern recognition receptors are an important player in the innate immune system. During the immune response to the hepatitis B virus, RIG-1 and TL3 play a significant role, and their functions might be impaired by the HBV polymerase [57]. Deficiency of TL3 in the placenta of HBsAg-positive mothers has been found to attenuate the vaccine response in infants, who are more vulnerable to chronic hepatitis infection, leading to deficiency in the cytokine levels in the innate immune system (IL-6, TNF-α, INF-α, and INF-γ, and Th1/Th2-specific (INF-γ, IL-4, and IL-10) cytokines), which are important in the immune response to the vaccine [58,59]. Other important pattern recognition receptors associated with the non-response are ficolin-2 and mannose-binding lectin, whose increases in concentration activate the lectin complement pathway [60]. This activation, conversely, results in bypassing the activity of the adaptive immune system and the development of immunological memory. Ficolin-2 and MBL also play a role in the pathogenesis of hepatitis B infection [61,62,63]. Mice with MBL deficiency produced a higher amount of hepatitis B antibodies than the wild-type mice, and when the MBL was reconstituted, the effect was abolished [64]. Also, a study from Indonesia revealed that a mutation of the MBL gene’s untranslated portion resulted in a non-response to the hepatitis vaccine [65]. Generally, within the innate immune system, elevated levels of proinflammatory and inflammatory genetic factors and cytokines at baseline predict a poor response to the hepatitis B vaccine, particularly in older individuals [66]. This was the first study to attempt to elucidate the mechanisms underlying non-response to the hepatitis B vaccine, especially at baseline.

#### 3.2.2. Involvement of Helper T Cells (Th)

CD4+ T cells play a significant role in the production of antibodies during infection [67]. This process after hepatitis B vaccination is tightly regulated by both Th1 and Th2 [68]. Specifically, Th2 responses are essential for high anti-hepatitis B antibody titres by promoting B cell activation, differentiation, class switching, and the formation of memory B cells, all of which are critical for robust and sustained immunity [69]. The action of these helper cells (cell-mediated cytolytic control and humoral production of specific antibodies by Th1 and Th2, respectively) is mediated by specific cytokines, including IL-2, TNF-γ, and TGF produced by Th1, and IL-4, IL-5, IL-6, IL-10, and IL-13 produced by Th2 [70]. In agreement, Sabry et al. [71] discovered a significant inhibition in overall Th1-specific cytokines, and Honorati et al. [72] observed the same for TNF-γ (specific to Th1) and IL-3 (specific to Th2) in hepatitis B vaccine non-responders. However, other conflicting findings indicated involvement of only Th2-specific cytokines (IL-4 and IL-5) and Th0 (mixed Th1/Th2), with no TNF-γ and IL-2 [73], while Böcher et al. (1999), Doedée et al. (2016), and Vingerhoets et al. (1995) asserted the involvement of only IL-4, IL-5, and IL-10, which are Th1-specific, contrary to the predominant involvement of Th2 cytokines [67,69,74]. Effects of both defective Th1 and Th2 were observed due to lower levels of a wider range of Th1 and Th2-specific cytokines, viz., INF-γ, TNF-α, IL-2, IL-4, IL-10, and IL-12, indicating a generalised dysfunction of T-cell-mediated immune response [75]. Moreover, the differentiation of B cells into memory cells and plasma cells that produce high-affinity antibodies is also mediated by T follicular helper cells (Tfh) through a reaction at the germinal centre. The reaction also involved the action of B6 cells, a transcriptional repressor, and IL-12 [76,77]. The main characteristics of the Tfh cells at the germinal centre are the expression of co-stimulatory molecules (ICOS), programmed death 1 (PD-1), and chemokine receptor 5 (CXCR5), which is also expressed by the circulating Tfh (cTfh) available in and more easily extracted from peripheral blood [78,79]. For research purposes, the cTfh is generally utilised, as it is also capable of releasing IL-12, which is necessary for the maturation of B cells, differentiation to produce plasma cells, and the release of antibodies [80]. To explore the role of post-transcriptional regulation of Tfh differentiation mediated by miRNA, Xu et al. [81] investigated the role of miR17-92 involved in the differentiation of Tfh, induced during T cell activation and suppression after differentiation of the T cells. It was found that antibody production is promoted by the miR-17-92 cluster, while miR-18a and miR-17 play a role in the development of Tfh cells following hepatitis B immunisation. Th17, which is another subset of CD4+ T cells, also plays a regulatory function similar to IL-10 mediated by IL-17 and IL-22, thereby serving a complementary effect with Treg cells [82]. Activation of macrophages, recruitment of Th1, B cell maturation, and differentiation, are all linked to the level of Th17 cells in circulation [83]. IL-22 has been linked to the cellular immune response to the hepatitis B vaccine [84]. Thus, IL-17 and IL-22 have a significant protective role to play during hepatitis B infection and in the immune response to vaccines. CD150 is another signal regulatory molecule for lymphocyte activation that influences the function of T cells, especially CD4+ T cells. It was more expressed in a non-responders cohort compared to responders in an Indian [85] and Chinese [86] population. Investigation into genetic polymorphisms on IL-17 identified the role played by the lower frequency of the rs4711998 GG genotype in a non-response and non-significant relationship with the rs22275503, rs2227501, and rs1026786 genotypes of IL-22 [87]. 

Further investigation into the role of deltex1, a protein located at the transmembrane portion of T cells, and its role in T cell anergy has been conducted [88,89]. Ten SNPs were identified, and two of them (rs2384077 and rs10744794), which are situated at the first deltex1 intron, had a remarkable association with the degree of immunological response among infant and adult Han populations in China. Although the role of introns in transcription remains insignificant compared to the coding regions, SNPs in the first introns were found to be involved in transcriptional functions, as were the exons [90].

#### 3.2.3. The Role of Regulatory B Cells

Regulatory B cells (Bregs) are a special category of cells in the immune system that inhibit the immune response through several mechanisms, which include activation of Treg cells, inhibition of CD+ Th cells, and inhibition of maturation and differentiation of plasma cells. This function is mediated through the regulation of the cytokine microenvironment, by the elevation of cytokines, such as IL-10, IL-35, and TGF-β, which have regulatory effects [91,92]. While the Bregs are known to be involved in several pathologies, such as cancer, multiple sclerosis, and systemic lupus erythromatosis [93], evidence shows variation in the elevated frequencies of two Bregs subpopulations, namely, CD24+/highCD27+ and CD24highCD38high, and enhanced IL-10 expression levels in hepatitis B non-responders [94,95]. The upregulated frequencies of CD24+/high CD27+ and CD24highCD38high were found to be reversed in non-responders, together with a lower expression level of IL-10 after receiving a booster dose with a recombinant third-generation vaccine for hepatitis B virus [96], which confirms the assertion by Bolther et al. (2018) [94] that the expression of IL-10-producing Bregs cells is not a good predictor of immunological response to the hepatitis B vaccination.

### 3.3. Genetic Variation in Hepatitis B Vaccine Non-Response

Variations in genetics have since been regarded as a major factor influencing the response to vaccination in more than 70% of non-responders [97], as vaccination of family relatives of known non-responders produces a higher non-response rate (58%) compared to the rate (5–10%) in the general population [98]. A study on gene expression identified about nine genes that were significantly upregulated in non-responders compared to responders, and these genes included nine coding RNAs, namely: transketolase-like 1 (TKTL1), carcino-embryonic antigen-related cell adhesion molecule 8 (CEACAM8), matrix metallopeptidase 8 (MMP8), bactericidal-permeability-increasing protein (BPI), folate receptor 3 (FOLR3), Defensin Alpha 1B (DEFA1B), Defensin Alpha 4 (DEFA4), Lactotransferrin (LTF), and transcobalamin 1 (TCN1), and the rest were non-coding genes involved in the regulation and expression process of CEACAM8. Three genes made of non-coding RNAs (a pseudogene and two long-non-coding RNAs) involved in the synthesis of proteins were downregulated [99]. These genes, which were present in the non-responding group, provided evidence of the presence of fixed genetic variations, which are independent of immunisation and could represent the transcriptome features of hepatitis B vaccine immune non-responders. miRNA-155, which is a small-non-coding RNA involved in several processes of immune response, was discovered at an increased amount in the serum of hepatitis B vaccine immune non-responders [100]. However, the non-response was more associated with SNPs in *miR-26a-1* and *miR-146a* of the miRNA [100]. On another note, granulin gene (GRN) expression was significantly upregulated in non-responders, indicating a greater activation state of the pathway for neutrophil activation in innate immunity. This greater GRN expression in non-responders likely results in the production of a higher amount of smaller granulins that are known to enhance the expression of Il-8 and the recruitment of more neutrophils. The IFITM1 gene, essential for immune response signalling, was downregulated at baseline in responders, indicating a state of greater immune system activation in the non-responders. However, after vaccination, IFITM1 was upregulated in the responders. The mechanism behind this observation remains to be elucidated [101]. Generally, the gene expression profiles of responders reflected the expected B and T cell responses to the vaccine. After vaccination, no peak of differentially expressed genes (DEGs) was observed in non-responders. Ten DEGs were noticed, among which three (KCTD7, DCAF12, and MARCH 8) are related to immune response and are involved in what is referred to as the ubiquitination process. Marked downregulation of KCTD7 in non-responders is interestingly difficult to interpret, as no differences exist between responders and non-responders for the other corresponding components (DCAF12 and MARCH 8) of the ubiquitination process [102]. According to these data, it can be said that in non-responders, only a small number of immune pathways are stimulated by the hepatitis B vaccine, and this occurred at different stages and rates than in responders.

#### 3.3.1. Antigen Processing and Presentation Genes

The immune response to vaccination involves the antigen processing and presentation to T cells, and antigen recognition by the T cells. The processing of the antigen and the presentation are mediated by the antigen-presenting cells (APC), containing a protein called the major histocompatibility complex (MHC). The communication that occurs between the APCs, B cells, and T cells is dependent on the function of surface molecules expressed on each of the cells, which include CD40, CD40L, CD25, and CD6 [103]. The genes on MHC DNA are important in the hepatitis B vaccine immune response, and it was found that the non-response gene is a recessive gene, which must exist in homozygous form to attenuate a response [104]. The first gene found to be responsible for the non-response was the [HLA-B8, SC01, DR3], especially in white people [105]. There is a significant heterogeneity in the expression levels of different HLA-I and HLA-II genes in antigen-presenting cells, particularly HLA-DRB5 and HLA-B, which are markedly downregulated [106]. This, in turn, affects antigen processing and presentation, as indicated in the “Kyoto Encyclopaedia of Genes and Genomes” (KEGG). Another role of the HLA was determined by assessing the influence of HLA-DPA1 and HLA-DPB1, where about nine alleles were found to be involved in keeping memory for the vaccine, which was determined after a booster vaccination [107]. Among the nine alleles, three were risk-associated while six were protective for the vaccine non-response [108]. The HLA-DPB1 alleles strongly related to non-response were determined to be at 05:01 and 09:01 after administering a booster vaccination in adolescent individuals with a history of active vaccination in the neonate period [107]. According to other findings aimed to provide more insight into the effect of genetic polymorphism in HLA-II alleles, DQB1-encoding β-portion on the antigen-binding groove of the DQ protein and DRB1 are also involved in the immune recognition and response. Ten different types of these alleles were found to be involved in the hepatitis B vaccine immune non-response [109]. DQB1*0401 was associated with non-response after vaccination with a three-dose schedule in a Japanese population [110]. Similarly, immune non-response was also associated with DRB1*07, according to studies from the USA [111] and Belgium [112], while a paradoxical finding was obtained in a study from Iran [113]. For DRB1*04, two studies from Taiwan [107] and Korea [114] provided evidence of its association with non-response. Conversely, a study in Germany involving pairs of twins associated DRB1*11 and DRB1*01 with a good immune response to the hepatitis B vaccination [115]. Another one from Taiwan also indicated that DRB1*08 favours a good response to booster vaccination. An investigation among the Chinese Han population identified a relationship between DRB1*14 and non- or hypo-response [116]. In summary, DRB1*01, DRB1*11, and DRB1*08, in contrast to DRB1*04 and DRB1*07, were in favour of non-response, as postulated in [117]. In a Japanese population, non- or hypo-response was significantly associated with HLA-DPB1*05:01, while a good response was associated with HLA-DPB1*04:02, HLA-DQB1*05:01, HLA-DRB1*01:01, and HLA-DRB1*08:03, as confirmed by the analysis of amino acid residues at different antigen-binding pockets of the HLA structural proteins [118]. The C4A locus of HLA class III was also found to be important, as it was downregulated in non-responders, especially HLA-C4AQ0, DRB1*0301, and DQB1*02 haplotypes, observed among non-responders and their close families [119,120]. These findings show that age, race, gender, and the nature of the virus may influence the functions of the genes involved. However, examining the influence of all these genes on the long-term immune response in those vaccinated early after birth revealed an insignificant relationship between these genes and the enduring immune response to the hepatitis B vaccination or booster vaccination [121]. Further genetic assessment of single-nucleotide polymorphisms (SNPs) by genome-wide association studies (GWAS) from different parts of the world, such as Japan, Thailand, China, Indonesia, and Korea, provided a holistic insight on the association of non-response with 10 different SNPs (rs2116260, rs3830066, rs5025825, rs35953215, rs7770370, rs6457709, rs35953215, rs3830066, rs7770370, rs3128961, rs9277535, rs9277542, and rs4282438) on loci HLA-DP. The strongest association was observed with rs7770370, which is also the major gene determining the response to booster dose, showing a significant effect on immunological memory in the long term [122]. Development of chronic hepatitis B infection and seroconversion was associated with rs9277535 and rs3077 on HLA-DP and rs2856718 and rs7453920 on HLA-DQ, in addition to their effect on non-response to hepatitis B vaccination [123,124,125,126].

#### 3.3.2. T Cell Regulation Genes

In hepatitis B vaccine immune non-response, the genes involved in the activity of naive and memory T cells, such as the MAPK signalling pathway and Nf-kb, are downregulated, as are the genes involved in many activities, such as cell activation, oxidative phosphorylation, interferon-γ, calcium ion reactions, and the IL-4 production of the three T-cell subsets, which are the CD4+ effector cells, CD8+ effector T cells, and CD8+ effector memory T cells. Meanwhile, cytotoxicity-associated biomarkers in NK and NKT cells are upregulated [106]. Genetic polymorphism in genes related to the function of Tfh cells also contributes to hepatitis B vaccine non-response, especially SNPs rs3922, affecting the expression of CXCR5 and rs355687 in CXCL13. Moreover, the allele “A” of the rs3922 reduces the expression of the CXCR5 more than the corresponding allele “G” due to the higher non-response observed with the “GG” genotype, followed by the “AG”, than “AA” [127].

#### 3.3.3. Genetic Polymorphism in Markers of the Immune System

Apart from the HLA genes, genetic polymorphism on the Th1 and Th2 cytokines and their receptors has largely been the focus of genomic research on hepatitis B vaccine non-response, owing to their essential contribution to the innate, cellular, and humoral immune systems. The effects of SNPs on various cytokines (IL-1β, IL4, IL4R, IL10, and IL12B) have been studied and reported in various populations from different parts of the globe [128,129,130]. For instance, the authors of [70] identified the role of IL12A and IL12B genes’ polymorphism in hepatitis B vaccine non-response. Also, a study on 53 SNPs on 21 different genes for toll-like receptors, cytokines, and their receptors, identified 4 SNPs (rs3804100 in TLR2, rs2243248 in the non-coding region of IL-4, rs1805015 in the coding region of IL-4RA, and rs1295686 in non-coding region of IL-13) strongly associated with non-response to the HBV vaccine [130]. Similarly, the INF-γ bioavailability and function are determined by the INFGR1 gene, whose genetic polymorphism, particularly the 170 and 182 allelic markers, is associated with hepatitis B vaccine immune non-response [131]. 

### 3.4. B Cell Amnesia-Associated Hepatitis B Vaccination Non-Response

**Figure 2:**
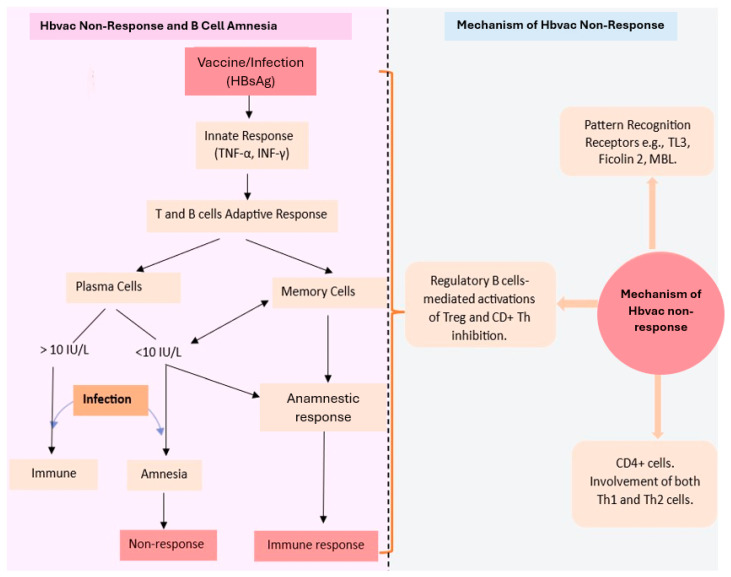
Diagram illustrating the concept of hepatitis B vaccine non-response and B cell amnesia and the mechanisms involved in the hepatitis B vaccine non-response. Hb_vac_: hepatitis B vaccine; HBsAg: hepatitis B surface antigen; TNF-α: tumour necrotic factor; α INF-γ: interferon γ; IU/L: international unit per litre; TL3: toll-like receptor 3; MBL: mannose binding lectin; Th: T helper; Treg: regulatory T cells.

The serum antibodies, despite being an essential measure for protection, are not the only indicators of successful immunisation. The generation of immunological memory is also an essential part of the immune response to vaccination [132]. The plasma cells generated during primary vaccination remain in the bone and produce antibodies constantly, while antibody detection in the serum long after vaccination is a marker for the activity of plasma cells that persist in circulation [133]. The plasma cells cannot detect antigens because they lack B cell receptors, which are present on the memory B cell surfaces. Thus, to induce the anamnestic response, B memory cells also generated from the germinal centre during the antigenic immune response are very necessary [134]. The memory B cells expressing a specific, high-affinity BCR upon encounter with their specific antigen, undergo differentiation into fresh plasma cells, and this accounts for the flare in high-affinity antibodies (IgG), typically described as an anamnestic response [135]. Some findings suggest independent regulation of plasma and memory B cells, hence the production of effective B cell memory in the absence of a defensive amount of anti-HbsAg antibodies [136,137]. Further proof of this is the non-progression to chronic infection by those in endemic areas without a sufficient level of antibodies after vaccination [138]. An examination of the distribution of specific lymphocytes identifies the same levels of memory B cells in both responders and non-responders, and the function of the memory B cells is maintained both in vivo and in vitro. Also, booster vaccination triggers no specific effect on the level of antibodies or memory B cell frequency [139]. However, in many cases, the memory B cell available in non-responders usually produces the same level of IgA and IgM, with a lower level of IgG, the detection of which depends on the type and sensitivity of the test method used [140]. This shows that even the non-responders, having supposed functional memory B cells, have a certain level of protection against chronic HBV infection [141]. The mechanism by which this happens remains an area for further research (Figure 2).

### 3.5. Strategies in Addressing HBV Non-Response

#### 3.5.1. Revaccination/Booster Dose

Revaccination in normal non-responders to vaccination requires careful consideration in selecting the type of vaccine to be used, as the nature of the vaccine, dose, and frequency of vaccination affect seroconversion in previous non-responders [142]. For example, in randomised controlled trials comparing the effectiveness of various brands of vaccine in previous non-responders, HBVaxPRO-40 and Fendrix produced a more significant response than Engerix, Twinrix, and HBVaxPRO-10, which necessitates expanding the indication for these vaccines or careful choice of a vaccine to enhance immunisation of real or suspected non-responders [143,144]. The revaccination of non-responders is also referred to as booster vaccination, in which a single higher dose (up to 60 µg) or an entirely new vaccination course of three doses is administered again [145,146]. Interestingly, the single higher dose has been found to have high immunogenicity and safety in several clinical trials [147]. After the booster dose, a long-lasting immunity is achieved when the anti-HbsAg level is greater than 100 IU/L [148]. A higher vaccine dose (or double dose) during the first immunisation may be used to achieve a better response in patients at risk of non-response conditions, such as chronic hepatitis C infection [149]. In non-responders with chronic kidney disease undergoing haemodialysis, revaccination is a common practice, in addition to using a higher (40 µg) vaccine dose at increased frequency due to the significant rate of non-response among them [150]. Meanwhile, a revaccination may be unnecessary in some perceived non-responders, as seroprotection may be delayed, as reported in a case report of a patient with haematological malignancy (a smouldering multiple myeloma) [151]. However, an experimental error in the laboratory, an HBV infection, or booster vaccination might be a risk factor. Demographic indices, such as age, gender, BMI, alcohol, and smoking, might also affect the response to revaccination, as reported in many studies [152].

#### 3.5.2. Use of Intradermal Route for Vaccine Administration

A change in the route of vaccine administration from the usual intramuscular route to the intradermal route has achieved considerable and comparable success in both the general population and special categories, such as people with chronic kidney disease, haemodialysis, celiac disease, and liver disease, though hypertension and advanced age limit the response in some of the categories [153]. As in celiac disease patients, a 40% higher response (anti-HbsAg ≥ 100 IU/L) was reported in booster vaccination through ID (Engerix B 2 μg), in contrast to IM (Engerix B 10 μg), in which only a 7.1% higher response was achieved [154]. In chronic liver disease patients, a significant response was achieved (≥10 IU/L in 70% of non-responders) with a high-dose intradermal vaccine (40 µg), without any increase in adverse events or dermatological reaction risk [155]. The reason for the enhanced response through the intradermal route compared to the intramuscular route could be the presence of dendritic and Langerhans cells, rich in MHC II molecules in human skin, warranting direct T cell activation, the release of inflammatory cytokines (especially IL-1) by keratinocytes that further enhance MHC-II expression on Langerhans cells, and the quick transfer of the antigen to the lymph node, where they persist for longer compared to IM, which is mostly localised [156,157]. This was also observed with vaccines for other infections, including influenza, smallpox, rabies, and polio [158]. Besides the enhanced immunogenicity, ID administration requires a lower vaccine dose and allows easy detection of immune responses, implying cost reduction, and recently, an enhanced delivery system using micro-needles [159]. However, most of the studies on the effectiveness of the ID route are not comprehensive, hence the need for studies that will focus on investigating the optimum dose, schedule of administration, and overall efficacy of the ID administration devices [160]. Meanwhile, a difference in response was also observed between administration through the gluteal muscle (yielding a lower response rate) and the deltoid muscle (yielding a higher rate of response) [161]. 

#### 3.5.3. Use of Adjuvants

Aluminium salts were the first adjuvants used to enhance the immune response of the body to the HBV vaccination. They act by eliciting the Th2-mediated response, leading to an increased rate of antibody production, and poorly inducing the Th1-cell-mediated response to enhance viral elimination [162]. The aluminium salts have undergone several modifications to improve their adjuvant action, including a strong capacity for adsorption, stability of suspension, and improved cell-mediated immune response [163]. Th1- (INF-γ) and Th2 (IL-13)-specific cytokines may be utilised as vaccine adjuvants to boost antibody responses [72]. Levamisole, IL-2, and granulocyte-macrophage colony-stimulating factor (GM-SF) are commonly employed as vaccine adjuvants in recombinant hepatitis B vaccines [164]. However, granulocyte colony-stimulating factor (G-CSF) was found to be more safe and effective than the GM-CSF, likely as a result of its restricted effect on only neutrophils, in addition to stimulation of antigen-presenting cells and dendritic cells capable of inducing Th2 lymphocytes [165]. Meanwhile, the efficacy of the GM-SF was better in chronic kidney disease [166] and HIV disease patients [167] than in healthy non-responders, in whom booster vaccination with a double dose of the recombinant vaccine produced a better response [168]. A hydrogel delivery system produced with a co-polymer (monomethoxy poly(ethylene glycol)-co-poly(lactic-co-glycolic acid (mPEG-PLGA) hydrogel) that is biodegradable and thermosensitive has been used to enhance the delivery of the GM-CSF together with the HbsAg at a local site, where activation of the immune system occurs through the activation of cytokines and dendritic cells in non-response mice strains [169]. Liposomes of cationic DC-Chol lipid have also been found to be more effective than aluminium hydroxide as an adjuvant in non-response mice, by enhancing cell-mediated immune responses [170]. Various other vaccine adjuvants have been formulated for specific categories of individuals with additional epitopes of viral proteins to enhance the immune response. PreS1/S2 envelope protein antigens are added to recombinant vaccines from the Chinese hamster ovary, referred to as third-generation hepatitis B vaccines, used for non-responders to conventional yeast-derived recombinant vaccines, immunosuppressed patients, overweight individuals, and patients with renal failure [171]. The third-generation vaccine has been proven to be more immunogenic by inducing more robust T and B cell immune responses, with an acceptable safety profile despite a few more side effects of injection site reaction, which was considered mild [172,173]. The immunomodulatory action of β-glycosphingolipid mediated through natural killer T cells (NKT cells) has been tested, and a positive effect was obtained, which further buttressed the role of the NKT cells in the HBV immune response [174]. For kidney transplant recipients, a single Fendrix^®^ dose, which is a third-generation vaccine containing the S and preS1/S2 antigens formulated with the adjuvant 3-O-desacyl-40-monophosphoryl lipid A (MPL), elicited a 60% response in previous non-responders after transplant, in addition to an enhanced hepatitis virus-specific cellular and humoral immune response [175]. This is comparable to the effect of conventional vaccines on healthy individuals. MPL adjuvant was also effective in patients with renal insufficiency who were previous non-responders, according to research carried out in Germany [176]. It was also formulated with Saponaria Molina to enhance the response after liver transplant [177]. These adjuvant vaccines are not usually used in healthy individuals due to concerns about side effects, difficulty in licensing highly immunogenic substances, and competing commercial interests among producers of conventional vaccines [178]. 

Another form of adjuvant is co-administration with other vaccines. The response to hepatitis B vaccines has been enhanced in non-responders and hypo-responders when administered together (in the same formulation) as a double dose with hepatitis A vaccine, in which a 90–95% response was achieved in previous non-responders [179]. The combined hepatitis B vaccine with the tetanus–diphtheria vaccine in dialysis patients who were non-responders also produced an enhanced but short-lived response [180], while the combination in healthy non-responders produced an enhanced but non-significant response compared to a single recombinant hepatitis B vaccine [181]. Another study compared the response of the combination in non-responders among pregnant women, haemodialysis patients, and healthy individuals that are non-responders and found that the response was enhanced more significantly in healthy non-responders compared to the other groups [182]. 

#### 3.5.4. Strategies in Chronic Kidney Disease (CKD) Patients

In CKD patients, especially those under haemodialysis, strategies employed include an increase in vaccine dose and frequency, addition of special adjuvants, revaccination or addition of a booster dose, and changes in the route of administering the vaccine [183,184,185,186]. Supplementation with vitamin D is also an effective enhancer of the immune response, in addition to the protection it affords to chronic kidney patients against hyperthyroidism and other conditions [187] (Table 1).

## 4. Discussion

Numerous factors, including the host immune status, defective immune cells, genetic variation, type, and nature of the vaccine, have been associated with non-response to the hepatitis B vaccine. The production and kinetics of cytokines from the peripheral blood mononuclear cells (PBMC) and their proliferation levels play a significant role in categorising individuals in relation to HBV vaccine response into high responders, low responders, non-responders, and naïve categories. The results of the present review indicate that non-response related to cell-mediated immunity is more CD4+-involved than CD8+, as hypothesised in an early report [188]. Meanwhile, the genetic mechanisms involved show that the non-response to the HBV vaccine involves a specific mechanism and does not indicate a general malfunction of the immune system, especially among healthy non-responders, where an adequate immunological response may be observed in the immune cells by stimulation with different antigens [189]. Also, in non-responders, despite the inadequate humoral response, there could be enough cellular response to protect them against the infection, as evidenced by the effect of the booster dose, revealing an anamnestic response. For genetic polymorphism, the majority of studies characterised non-responders as having specific alleles that are not present in responders, showing that the non-response alleles are markers for the genetic risk of immune pathologies, such as celiac disease, diabetes, and inflammatory bowel disease, and vice versa. 

Strategies to curtail non-response include revaccination or booster vaccination, the use of adjuvants, and changing the route of administration. These strategies are sometimes influenced and determined by the nature of HLA haplotypes in the recipients. A proper understanding of the mechanisms and factors involved in the non-response significantly influenced the development of means and strategies to prevent the non-response, thereby reducing the burden of chronic hepatitis B infection. The mechanisms of non-response largely influenced by genetic polymorphism vary substantially among different populations around the globe, which necessitates a comprehensive understanding to allow proper implementation of the various prevention strategies. There seems to be a great paucity of information about the mechanisms of non-response from the West African populations, who are among the high pandemic areas with high vulnerability to chronic HBV infection and a poor healthcare system for effective management of the disease [190]. From the foregoing, B cell amnesia remains an area in need of further research to determine its cause and effect as it relates to hepatitis B vaccine non-response.

In conclusion, the non-response to the hepatitis B vaccine can be said to be multi-specific and polyclonal, therefore requiring a comprehensive understanding and systematic approach to mitigate it. This will facilitate the achievement of the WHO 2030 goal of achieving 90% global immunisation, a prevalence reduction to 0.1%, and an 80% improvement in treatment. Although the vaccine proves effective significantly, its role in the program needs to be reevaluated considering the various limitations discussed in this review.

## Figures and Tables

**Table 1:** Some common hepatitis B vaccines and their key features.

Characteristic	HBVaxPRO-40	Fendrix	Engerix	Twinrix	HBVaxPRO-10	Third-Generation Vaccines
**Effectiveness in Non-responders**	High	High	Moderate	Moderate	Moderate	High
**Dose**	Up to 60 µg (booster)	40 µg (chronic kidney)	Variable (10 µg standard)	Variable (combination)	10 µg	S, preS1/S2 antigens
**Frequency**	Booster/three doses	Increased frequency	Standard/booster doses	Standard/booster doses	Standard/booster doses	Variable (dependent on patient condition)
**Administration Route**	Intramuscular	Intramuscular	Intramuscular	Intramuscular	Intramuscular	Intramuscular or with adjuvant systems
**Special Conditions**	High immunogenicity	High response in kidney disease	Moderate response in general population	Moderate response in general population	Moderate response in general population	Enhanced immunogenicity in specific conditions
**Use of Adjuvants**	No	Yes (MPL)	No	No	No	Yes (e.g., GM-CSF, MPL, liposomes, PreS1/S2 proteins)
**Side Effects**	Mild to moderate	Mild	Mild	Mild	Mild	Mild injection site reactions
**Additional Notes**	Suitable for higher-risk non-responders	Effective in chronic kidney disease	Commonly used; standard vaccine	Combination vaccine (Hepatitis A and B)	Commonly used; standard vaccine	More robust T and B cell responses, used in immunocompromised patients

## Data Availability

Not applicable.
